# Tapping onto the Potential of Smartphone Applications for Psycho-Education and Early Intervention in Addictions

**DOI:** 10.3389/fpsyt.2016.00040

**Published:** 2016-03-17

**Authors:** Melvyn W. B. Zhang, Roger C. M. Ho

**Affiliations:** ^1^Biomedical Global Institute of Healthcare Research & Technology (BIGHEART), National University of Singapore, Singapore, Singapore; ^2^Department of Psychological Medicine, National University Healthcare Systems (NUHS), Singapore, Singapore

**Keywords:** mental health, addiction, psychiatry, smartphone, mobile phone, E-health, m-health

## Abstract

E-health, and in particular smartphone-based technology, is increasingly becoming commonplace in healthcare. While psychiatry has tapped onto these innovations for conditions, such as affective disorders, and schizophrenia and psychosis, the usage of these technologies in addiction is limited. Addiction psychiatry could harness the potential of smartphone technologies. Given the increasing incidences of substance-related problems globally, and along with the normalization of the general public’s perspectives toward substances, and also in consideration of unwillingness for at-risk individuals in seeking help, the authors hope to illustrate how these issues could potentially be solved using E-health and technological innovations. The objectives of the current perspective article are to illustrate how recent advances in smartphone-based technologies could help in terms of psycho-education, as well as in helping individuals who are at-risk users in seeking help earlier. The authors aim to illustrate how the above are possible, building on existing theory-driven framework that has been extensively reviewed in the previous literature. Limitations with regard to the implementation of such technologies will also be discussed.

## Perspectives

Zhang et al. (1) have highlighted in their recent article, how psychiatrists could be empowered in harnessing the full potential of E-health. Globally, E-health has affected the way healthcare professionals work. With the implementation and the integration of E-health into the existing healthcare framework, it has certainly improved the efficacy of work processes. E-health in itself encompasses a wide variety of technological advances, which include that of telemedicine, mobile devices, and their accompanying applications, as well as wearable devices. Over the last decade, there has been major development and growth in terms of the development of healthcare-related smartphone applications. With the launch of these smartphone applications, individuals are now able to be in control and are now able to self-manage their own conditions using their smartphone. In the field of psychiatry, there are smartphone innovations such as smartphone applications that could help to enforce medication compliance for schizophrenic patients (2). Other smartphone applications could also facilitate the self-reporting of symptoms among schizophrenia patients (3). This would enable psychiatrists to be better aware of how their patients are progressing and the stressors that their patients might be experiencing that might trigger a relapse of their underlying conditions (3). The application of technology is not limited to schizophrenia in psychiatry, but it does extend to other mood and affective disorders (4) and also substance disorders (5). Zhang et al. (1) have previously recommended two simple modalities to which psychiatrists could tap onto and harness the potential that technology has brought into healthcare. It is also important for psychiatrists to recognize the limitations of psychiatry-related application out there in the smartphone application marketplace and be able to recommend evidence-based applications to their patients. Hence, Zhang et al. (1) recommended that psychiatrists ought to use validated scales, such as the Silberg scale (6), to help them to evaluate the information quality of the existing applications. In addition, the authors (1) have also pointed out another scale, known as the mobile application rating scale (7) that has just been developed, that might be suitable for identification of applications with good evidence base. On knowing the current limitations of psychiatry-related smartphone applications in the marketplace, psychiatrists could then play a part in the conceptualization and development of smartphone innovations that not only help to mitigate the current gaps in the existing applications but also help in the conceptualization of applications that are in-line with the needs of their profile of patients (1). More importantly, psychiatrists could help in the conceptualization and development of applications that are grounded in theory and in accordance to established behavioral change models. Subhi et al. (8) have previously shared how smartphone applications could be built using just a text editor alone. However, Zhang et al. (9, 10) have highlighted the limitations of that particular methodology, and have shared their methodologies of overcoming those limitations. In particular, they have highlighted how the usage of an online application builder could help in the development of low-cost interactive applications, which could eventually be deployable to the various smartphone application stores, for ease and convenience of download.

To date, one of the most challenging issues to deal with in psychiatry currently has been the increasing incidence of substance and drug abuse and dependence. The increasing incidences of such abuse and dependency are of concern, as these disorders do predispose vulnerable individuals to other comorbid psychiatric disorders, such as affective disorders and psychosis. There are a multitude of reasons for the increasing incidences globally, but one of the likely causative factors might be due to the normalization of the general public’s perspective toward drugs of abuse. Following the legalization of marijuana or cannabis in Colorado (11), it was found that there have been increases in the incidences of individuals in Colorado who have presented themselves to the emergency departments due to an acute intoxication of commonly abused substances. The availability of marijuana in the forms of edible products, such as muffin, would serve to further reinforce the idea that marijuana is safe for once-off usage and consumption. The availability of these products does have its accompanying dangers as well, not just in terms of the risk of abuse and addiction. Vulnerable individuals, such as children, might consume them unknowingly. Of note, in Colorado (12), there has been a growing incidence of children who have presented to the emergency services following an acute intoxication of these substances. It does not help that with the legalization of marijuana, perspectives toward substances have shifted. There are on-going clinical trials involving ketamine and ecstasy (MDMA), and these trials have purported that ketamine does have inherent rapid antidepressant efficacy, while MDMA would facilitate psychotherapy for individuals with post-traumatic stress disorder (13, 14). Researchers have previously highlighted how the mass media has hyped up the clinical efficacy of these drugs, such as ketamine (15), and this will further cause a mass media effect and lead to normalization of perception toward drugs and substance usage. Despite the promising trials conducted initially, there has been extensive evidence released recently by the Cochrane collaboration (16, 17) of the limited efficacy of ketamine in the treatment of depression.

Addiction specialists or even psychiatrists who have worked with individuals with dual diagnosis (with addiction as a co-diagnosis) know that dealing with substance-related issue is particularly tough. Most individuals with substance-related issues as their primary problems do not routinely seek help. There has been much literature supporting this locally and overseas. Rehm et al. (18) in recent cross-sectional study highlighted that, in comparison to other mental health disorders, substance use disorder has the lowest treatment rates, despite them having the highest burden in terms of morbidity and mortality. There have been a multitude of reasons accounting for rates. The World Health Organization purported that it was in part due to the failure of primary care physician in terms of recognition of these disorders. Stigmatization mediates the help-seeking relationship and the last reason being that these individuals tend to prefer not to seek help unless they have hit rock-bottom (18).

With the advances in smartphone technologies, there have since been a lot of applications that have been developed for addiction. Previous content analysis done for Marijuana-related applications (19) has highlighted the concern about the evidence base and the information quality of the existing applications on the store. Most of the applications are for entertainment purposes, and most of these applications do not provide accurate information about the dangers associated with the usage of Marijuana (19). Another prior content analysis (20) done for alcohol-related application highlighted the fact that most of the current alcohol applications made available in the store attempt to track the amount that an individual has drunk using the blood alcohol concentration method (20). The content analysis highlighted that such methodology might results in individuals drinking even more, as an attempt to challenge their pre-set limits (20). A keyword search of the existing research literature online reveals that there are to date several published literature about how technology could help in substance-related disorders. Prior review done by Keoleian et al. (21) has looked at the efficacy of the utilization of text messaging for addiction. The 11 studies (which included smoking, alcohol, and marijuana and methamphetamine addiction) that they have looked at have highlighted that text messaging is effective in helping individuals in achieving abstinence. For alcohol addiction, prior research has demonstrated that smartphone-based brief interventions could help targets drinking choices amongst University students (Gajecki et al.) (22). ACHESS, another smartphone application to support patients in their recovery from alcoholism has its basis on self-determination theory (McTavish et al.) (23). The ACHESS smartphone application helps to prevent users from heavy drinking post treatment, using features such as geo-location services to locate individuals when they are close to geographically high-risk locations. Other functionality implemented includes that of a mood and a withdrawal questionnaire and charting. LBMI-A (24) is another application for intervention for alcohol issues and provides individuals with assessment, information, and intervention.

The objectives of the current perspective article are to illustrate how recent advances in smartphone-based technologies could help in terms of psycho-education, as well as in helping individuals who are at-risk users in seeking help earlier. The authors aim to illustrate how the above ones are possible, building on existing theory-driven framework that has been extensively reviewed in previous literature. Limitations with regard to the implementation of such technologies will also be discussed.

## Smartphone as Psycho-Educational Tools

Prior research has described how smartphones could educate individuals who are diagnosed with HIV in risk reduction (25). More recent research has demonstrated the utility of web-based alcohol screening and brief intervention tools for University students (26). The literature review above demonstrated that there are existing applications that provide information about substances. While there might be existing interventions designed to educate the general public about substances, it is concerning that most of the other applications on the application stores do not provide the similar quality of information, in accordance with the findings of the previous content analysis of existing applications. Addiction psychiatrists could, thus, play a further role in the conceptualization and the development of new applications that aims to provide further information about substances, much like what the Royal College of Psychiatrists in the United Kingdom has done. Rosenbaum (27) have proposed that in order for education about drugs to be successful in today’s world, taking into consideration the recent drive toward legalization, it is thus of importance that programs developed have a scientific basis, and that these programs would encourage moderation of usage. In addition, apart from the provision of scientific information, it is pertinent now for applications to include relevant legal statues and laws to deter further experimentation.

Prior to the introduction of the web as well as smartphone technologies, the mass media has been utilized for various substance-related problems. The Cochrane collaboration (28) previously evaluated mass media interventions for smoking cessation and has concluded that these interventions are deemed to be effective in smoking cessation. Mass media interventions include that of the television, radio, newspapers, billboards, and also posters and leaflets. Other studies (29) have also demonstrated the utility of videos in encouraging individuals to adopt healthy behaviors, such as going for HIV testing. The Royal College of Psychiatrists in the United Kingdom has always been a strong advocate for psycho-education of the general public to increase public’s literacy about common mental health disorders. The previous mechanism of delivery of such materials was via their online website as well as printed brochures. Recently, the college has tapped onto smartphone technologies and has launched the RCPsych Mental Health Application to provide members of the general public with easier access to a smartphone friendly version of this information. The conceptualization and the development of the application are led by the first author, making use of the methodologies previously described by Zhang et al. (9, 10). The newly developed version of the smartphone application has not only included the traditional print materials but the smartphone application has also included animations and podcasts in order to provide users with alternatives to gather the information that they require.

**Figure 1:**
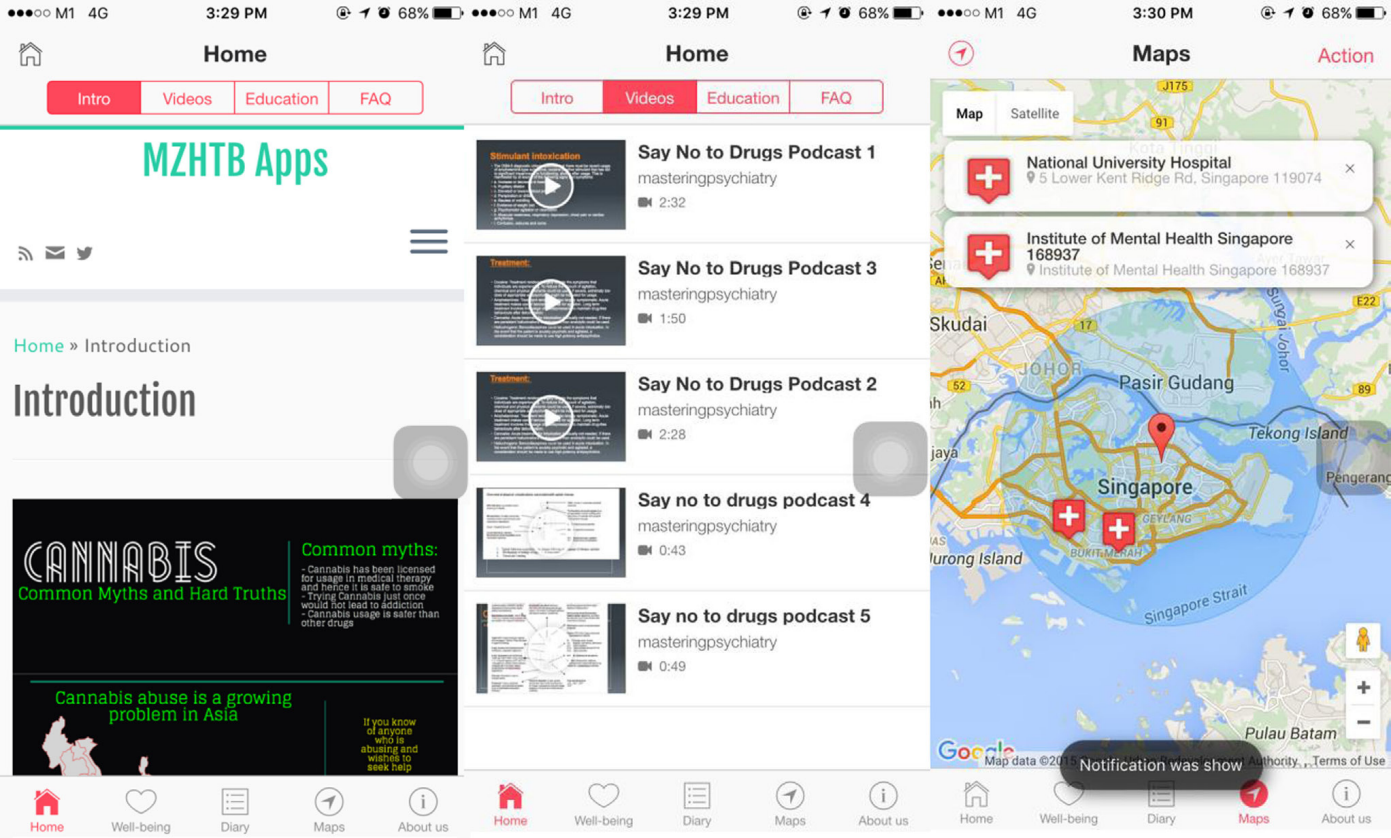
**Overview of the conceptualization of a Drug and Substance Psycho-education Program**.

Thus, taking into consideration the limitations of existing applications on the store, and the recent recommendations by Rosenbaum (27), and also taking into consideration how prior mass media interventions have been deemed to be useful in promoting health behaviors, the authors hope to share their conceptualization of a smartphone-based intervention that is based on existing theories. The methodologies described previously by Zhang et al. (9, 10) are limited in the sense that those methodologies described do not enable the addition of interactive features into smartphone applications. In addition, there are inherent difficulties with the publication of these applications to the application stores. It is of importance for these applications to be featured on the stores, so that it is more accessible for the general public to download. With further advances in technology, there are more strategies that could enable psychiatrists to create evidence-based application that are in-line with what they feel patients and the general public might benefit from. Apache Cordova (30) is one such potential method, which could enable psychiatrists to create cross-platform compatible application. Given the inherent effectiveness of videos, it is thus envisioned that in the current application, there will be integration of animation, info graphics as well as videos to better communicate key messages about drugs to individuals. There should also be other interactive features, such as that of a geo-location service that would be able to locate the precise location of the user. If users are intoxicated after using drugs, they would be able to tap onto the application to look for the nearest medical facility to receive treatment from. This conceptualization is based on the theory-driven model of identification of high-risk locations for alcohol addiction, as previously described. Figure 1 illustrates the core features of an evidence-based drug psycho-education smartphone application.

## Smartphone Interventions for At-Risk Users

As discussed previously, one of the key challenges in addiction psychiatry is that the vast majority of individuals do not seek help for their issues. Various studies have highlighted various reasons to account for this. Harm reduction has been widely looked into and is currently one of the intervention strategies in addiction psychiatry. Logan and Marlatt (31) have proposed harm reduction as one of the intervention strategy for youths who are at-risk for addictive behaviors, such as alcohol addiction. With regard to the problem of at-risk drinking, prior studies have also demonstrated the superiority of controlled drinking over abstinence approaches (Marlatt and Witkiewitz) (32). With the recent legalization of substances, such as marijuana, and with the push for the usage of illicit substances, such as ketamine and MDMA for psychiatric treatment, there might be normalization of the perspectives held by the general public toward drugs. It is important to target those who are at-risk users early, as the earlier an intervention is implemented, the better the resultant prognosis of the underlying condition.

As reviewed previously, most of the current literature showed that to date most of the interventions are geared toward supporting the recovery process of patients with addiction issues. While there is a wide variety of smartphone-based applications out there in the application stores, the previous content analysis have clearly highlighted the drawbacks of such applications, in that the majority of them are lacking in terms of evidence base. Given that the objective of this article is to highlight how smartphone technologies could be applied in the domain of addiction psychiatry and in particular, applied for at-risk individuals, using theory-driven framework, the authors would describe two smartphone applications that they have conceptualized and developed. The theoretical framework underpinning these interventions is that of harm reduction.

The alcohol tracker application has been previous described by Zhang et al. (34). The currently available alcohol trackers on the application store attempts to minimize harm among at-risk drinkers through enabling them to self-monitor their blood alcohol concentration. Some applications do not seemed to help reduce drinking as it promotes and encourages users to challenge their pre-defined limits. Thus, instead of tracking the absolute amount of drinks that an individual has had and converting it to a blood alcohol concentration, and advising the individual when it would be safe for them to engage in their routine activities, such as driving again, the conceptualization by Zhang et al. (34) attempts to empower individuals in helping them to track the amount that they have drank and warning them if they are over the limits as stipulated by the respective guidelines. The alcohol tracker application conceptualized (34) makes no recommendation of the timing to which individuals are deemed safe to return to their original activities. In addition, in order to ensure that individuals who are deemed to be at-risk would be able to receive help, the alcohol tracker application incorporates information not only about alcohol abuse and dependence but has also links to a direct line to which individuals could call (if they are in Singapore) and they will be advised about how best to receive help. Despite the fact that the conceptualization of the application is done in Singapore, much of the application is relevant for an international audience. There are not much variant in terms of the recommended guidelines with regard to the number of units individuals are to consume in a day and a week, and the information of alcohol abuse and dependence and adverse medical risks associated with alcohol also does not differ globally. Collaborators from the University of British Columbia were, thus, invited to help evaluate the conceptualized applications and to gather user perspectives with regard to the new conceptualization, that has a theoretical basis (34). A group of 100 Canadians were recruited in order to gather user perspectives. The results obtained showed that notification and information were regarded as the most useful functions of the innovative application (34).

The alcohol tracker application is just one of the two smartphone innovations that the authors wish to highlight that could potentially help individuals who are at-risk. The only smartphone-based intervention for gamblers seemed to be limited to an online gambling self-help workbook currently, which has its theoretical basis on harm reduction and behavioral strategies (35). Users are asked to identify high-risk situations and provided with personalized feedback with regard to dealing with these situations; and, in addition, they are also able to compute their financial risks (35). In order to help at-risk gamblers, the authors have jointly conceptualized the eGambling Intervention application (36). The eGambling Intervention smartphone application might seem that it is only capable of providing general information about gambling addiction and how best to seek help and treatment. However, the main developer (MWBZ) has actually made use of the build in global positioning sensor in the smartphone to track the position of the individual. The locality and the position of the individual would be tracked and with the coordinates updated once every 20 s and feedback to a server. Once the individual is within the proximity of the 25 gambling locations in Singapore, a notification is sent to them. The number of notifications sent and the duration will be tracked and sent back to an Apache Server. In addition, there is the enhanced functionality of allowing individuals to phone their family members or even their counselors to get help. If they chose not to phone, they could also get help by means of an email notification. Thus, by means of the application, the authors are able to determine the approximate number of visits individuals make to the casinos to gamble. One of the potential limitations is that the authors have not tapped onto the other sensors, in order to understand more about the individual’s behavior prior to the gambling episode. The integration of other sensors to track the individual’s behavior prior to the activity would open up opportunities for ecological momentary analysis. While the application does not allow for ecological monetary analysis currently, the theoretical framework underpinning this intervention is once again that of harm reduction, in preventing users from frequenting casinos; as well as behavioral approaches that have been previously described in the literature, by enabling users to identify high-risk locations and providing interventions accordingly.

**Figure 2:**
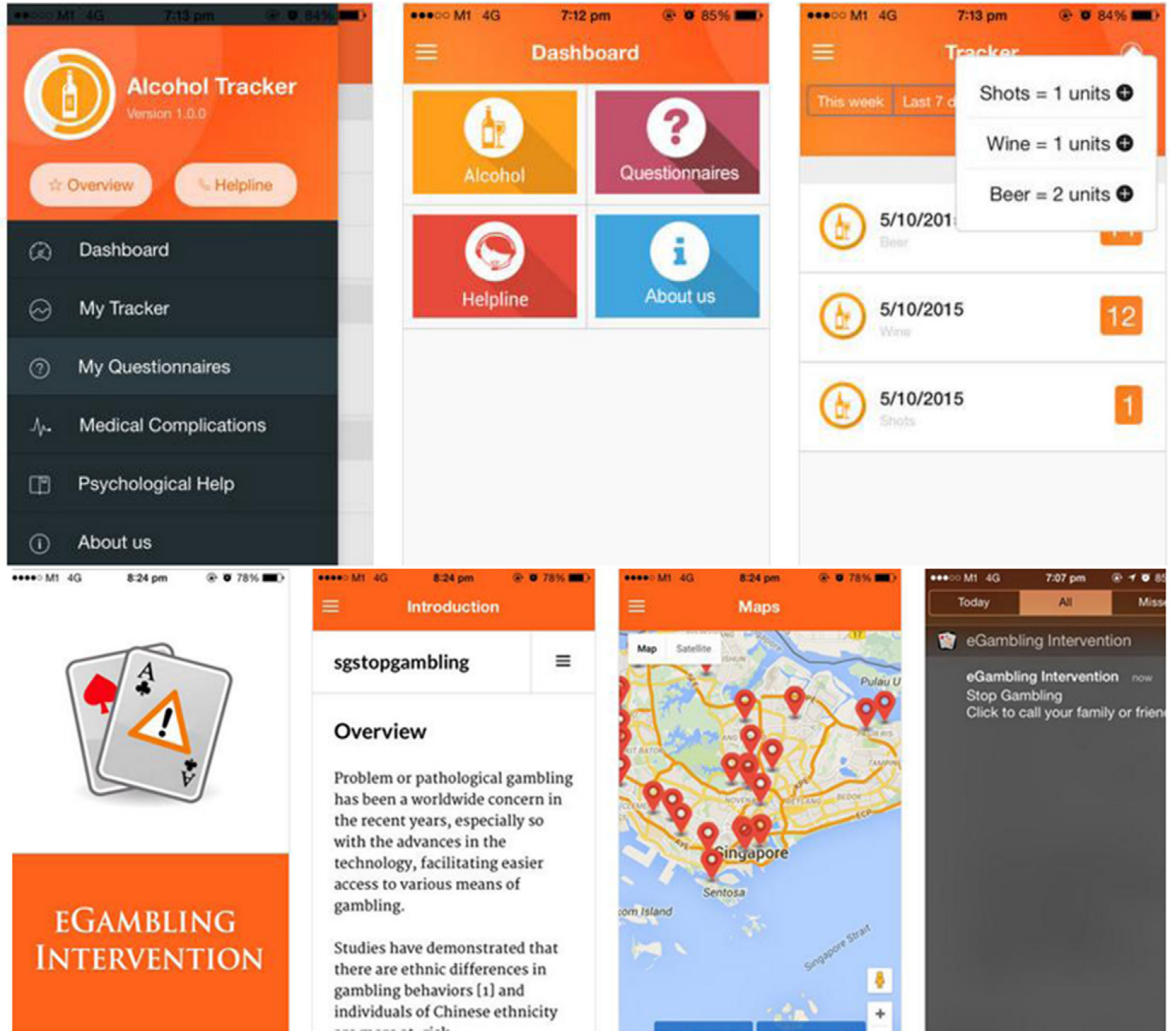
**Overview of the conceptualization and features of an alcohol tracker application and a gambling intervention for at-risk individuals**.

Figure 2 illustrates the core features of each of the application.

## Potential Limitations and Ethical Concerns

Smartphone applications might seemingly be the answer and solution for addiction-related psychiatric problems. However, it is still key to recognize that they are certainly not a replacement for a consultation with a mental health specialist. The assessment that applications could do is limited. In addition, smartphone applications might not reach out to those who do not have access to smartphones and their accompanying applications, or who have no knowledge with regard to how best to use smartphone applications. While psychiatrist could help in the conceptualization and development of the applications, it is still key for applications to be continuously kept updated, even after publication. In addition, gathering patient information on smartphone devices raises concerns about ethics and security.

## Conclusion

E-health, and in particular smartphone applications, is increasingly becoming commonplace in healthcare. While psychiatry has tapped onto these innovations for conditions, such as affective disorders, and schizophrenia and psychosis, the usage of these technologies in addiction is limited. Addiction psychiatry could harness the potential of smartphone technologies in educating the masses about the harmful effects of drugs. This is particularly important given the changing perception held by individuals toward commonly abused drugs, as more drugs are being legalized or might be legalized for medical usage. Smartphone technologies incorporating theory-driven framework could be harnessed and used as interventional tool for those who are at-risk for the development of addiction. However, there remain limitations to the usage of such technologies that should be carefully considered.

## Author Contributions

All authors have contributed equally to the manuscript.

## Conflict of Interest Statement

The authors declare that the research was conducted in the absence of any commercial or financial relationships that could be construed as a potential conflict of interest.

## References

[1] Zhang MW Ho RC Enabling psychiatrists to explore the full potential of E-health. Front Psychiatry (2015) 6:177 10.3389/fpsyt.2015.00177 26696912 PMC4678871

[2] Granholm E Ben-Zeev D Link PC Bradshw KR Holden JL Mobile assessment and treatment for schizophrenia: a pilot trial of an interactive text-messaging intervention for medication adherence, socialization and auditory hallucinations. Schizophr Bull (2011) 38(3):414–25. 10.1093/schbul/sbr155 22080492 PMC3329971

[3] Palmier-Claus JE Ainsworth J Machin M Barrowclough C Dunn G Barkus E The feasibility and validity of ambulatory self-report of psychotic symptoms using a smartphone software application. BMC Psychiatry (2012) 12:172. 10.1186/1471-244X-12-172 23075387 PMC3502449

[4] BinDhim NF Sharman AM Trevena L Basyouni MH Pont LG Alhawassi TM Depression screening via a smartphone application: cross-country user characteristics and feasibility. J Am Med Inform Assoc (2015) 22(1):29–34. 10.1136/amiajnl-2014-002840 25326599 PMC4433364

[5] Rizvi SL Dimeff LA Skutch J Carroll D Linehan MM. A pilot study of the DBT coach: an interactive mobile phone application for individuals with borderline personality disorder and substance use disorder. Behav Ther (2011) 42(4):589–600. 10.1016/j.beth.2011.01.003 22035988

[6] Eysenbach G Powell J Kuss O Sa ER. Empirical studies assessing the quality of health information for consumers on the world wide web: a systematic review. JAMA (2002) 287(1):2691–700. 10.1001/jama.287.20.2691 12020305

[7] Stoyanov SR Hides L Kavanagh DJ Zelenko O Tjondronegoro D Mani M. Mobile app rating scale: a new tool for assessing the quality of health mobile apps. JMIR Mhealth Uhealth (2015) 3(1):e27. 10.2196/mhealth.3422 25760773 PMC4376132

[8] Subhi Y Todsen T Ringsted C Konge L. Designing web-apps for smartphones can be easy as making slideshow presentations. BMC Res Notes (2014) 7:94. 10.1186/1756-0500-7-94 24552200 PMC3931664

[9] Zhang M Tsang T Cheow E Ho C Beng Yeong N Ho R. Enabling psychiatrists to be mobile phone app developers: insights into app development methodologies. JMIR Mhealth Uhealth (2014) 2(4):e53. 10.2196/mhealth.3425 25486985 PMC4285745

[10] Zhang M Cheow E Ho CS Ng BY Ho R Cheok CC. Application of low-cost methodologies for mobile phone app development. JMIR Mhealth Uhealth (2014) 2(4):e55.1. 10.2196/mhealth.3549 25491323 PMC4275474

[11] Monte AA Zane RD Heard KJ The implications of marijuana legalization in Colorado. JAMA (2015) 313(3):241–2. 10.1001/jama.2014.17057 25486283 PMC4404298

[12] Hurley W Mazor S Anticipated medical effects on children from legalization of marijuana in Colorado and Washington state: a poison centre perspective. JAMA Pediatr (2013) 167(7):602–3. 10.1001/jamapediatrics.2013.2273 23712729

[13] Sessa B Nutt D. Making a medicine out of MDMA. Br J Psychiatry (2015) 206(1):4–6. 10.1192/bjp.bp.114.152751 25561485

[14] Young MB Andero R Ressler KJ Howell LL. 3,4-Methylenedioxymethamphetamine facilitates fear extinction learning. Transl Psychiatry (2015) 5:e634. 10.1038/tp.2015.138 26371762 PMC5068803

[15] Zhang MW Ho RC Ketamine’s potential as a rapid antidepressant was overplayed. BMJ (2015) 351:h4467 10.1136/bmj.h4467 26290499

[16] McCloud TL Caddy C Jochim J Rendell JM Diamond PR Shuttleworth C Ketamine and other glutamate receptor modulators for depression in bipolar disorder in adults. Cochrane Database Syst Rev (2015) 9:CD011611. 10.1002/14651858.CD011611.pub2 26415966

[17] Caddy C Amit BH McCloud TL Rendell JM Furukawa TA McShane R Ketamine and other glutamate receptor modulators for depression in adults. Cochrane Database Syst Rev (2015) 9:CD011612. 10.1002/14651858.CD011612.pub2 26395901

[18] Rehm J Manthey J Struzzo P Gual A Wojnar M. Who receives treatment for alcohol use disorders in the European Union? A cross-sectional representative study in primary and specialized health care. Eur Psychiatry (2015) 30(8):885–93. 10.1016/j.eurpsy.2015.07.012 26647862

[19] Ramo DE Popova L Grana R Zhao S Chavez K. Cannabis mobile apps: a content analysis. JMIR Mhealth Uhealth (2015) 3(3):e81. 10.2196/mhealth.4405 26268634 PMC4705020

[20] Emma RW Danielle RH Rebecca J Paul D Megan SCL “Let’s get wasted!” and other apps: characteristics, acceptability, and use of alcohol-related smartphone applications. JMIR Mhealth Uhealth (2013) 1(1):e9 10.2196/mhealth.2709 25100681 PMC4114432

[21] Keoleian V Polcin D Galloway GP. Text messaging for addiction: a review. J Psychoactive Drugs (2015) 47(2):158–76. 10.1080/02791072.2015.1009200 25950596 PMC4537651

[22] Gajecki M Berman AH Sinadinovic K Rosendahl I Andersson C. Mobile phone brief intervention applications for risky alcohol use among university students: a randomized controlled study. Addict Sci Clin Pract (2014) 9:11. 10.1186/1940-0640-9-11 24985342 PMC4091647

[23] McTavish FM Chih MY Shah D Gustafson DH. How patients recovering from alcoholism use a smartphone intervention. J Dual Diagn (2012) 8(4):294–304. 10.1080/15504263.2012.723312 23316127 PMC3541672

[24] Dulin PL Gonzalez VM Campbell K. Results of a pilot test of a self-administered smartphone-based treatment system for alcohol use disorders: usability and early outcomes. Subst Abus (2014) 35(2):168–75. 10.1080/08897077.2013.821437 24821354 PMC4019942

[25] Phillips KA Epstein DH Mezghanni M Vahabzadeh M Reamer D Agage D Smartphone delivery of mobile HIV risk reduction education. AIDS Res Treat (2013) 2013:231956. 10.1155/2013/231956 24159383 PMC3789326

[26] Kypri K Vater T Bowe SJ Saunders JB Cunningham JA Horton NJ Web-based alcohol screening and brief intervention for university students: a randomized trial. JAMA (2014) 311(12):1218–24. 10.1001/jama.2014.2138 24668103 PMC4413370

[27] Rosenbaum M New perspectives on drug education/prevention. J Psychoactive Drugs (2016) 2:1–3. 10.1080/02791072.2015.1117690 26799842

[28] Bala MM Strzeszynski L Topor-Madry R Cahill K. Mass media interventions for smoking cessation in adults. Cochrane Database Syst Rev (2013) 6:CD004704. 10.1002/14651858.CD004704.pub3 23744348

[29] Tuong W Larsen ER Armstrong AW. Videos to influence: a systematic review of effectiveness of video-based education in modifying health behaviors. J Behav Med (2014) 37(2):218–33. 10.1007/s10865-012-9480-7 23188480

[30] Apache Cordova. (Last assessed on 8th March 2016). Available from: https://cordova.apache.org

[31] Logan DE Marlatt GA. Harm reduction therapy: a practice-friendly review of research. J Clin Psychol (2010) 66(2):201–14. 10.1002/jclp.20669 20049923 PMC3928290

[32] Marlatt GA Witkiewitz K. Harm reduction approaches to alcohol use: health promotion, prevention, and treatment. Addict Behav (2002) 27(6):867–86. 10.1016/S0306-4603(02)00294-0 12369473

[33] Zhang MW Ho RC The alcohol self-management smartphone application: an evidence based approach. BMJ Innov (2015) 1:74 10.1136/bmjinnov-2015-000057

[34] Zhang MWB Ward J Ying JJB Pan F Ho RCM The alcohol tracker application: an initial evaluation of user preferences. BMJ Innov (2016) 2:8–13. 10.1136/bmjinnov-2015-000087 PMC4789684 27019744

[35] Zhang MW Yi Y Cheok CC. Internet based personalized feedback interventions for gamblers in Singapore: first results. Technol Health Care (2015). 26578285 10.3233/THC-151117

[36] eGambling Intervention. (Last assessed on March 8th, 2016). Available from: https://play.google.com/store/apps/details?id=com.melvyn.stop_gambling&hl=en

